# Insanity Treated

**Published:** 1867-10

**Authors:** 


### INSANITY TREATED.

POOR Carlotta, the amiable, faithful and accomplished wife of MAXIMILIAN, who acknowledged a short time before his execution that if he had followed her advice he would have avoided the sad fate which befel him, has the sympathy of the entire civilized world. Two plans have been proposed by two eminent medical men for restoring her to her reason. One was to take her to the home of her childhood and surround her with all the circumstances which would be calculated to wake up her attention and carry her back to the happy home of her joyous youth. They were even so particular as to search two kingdoms to find a little pony which should be a fac-simile to the one she so much delighted to ride in childhood. One was found in size, age, gait, color and everything, in a Frenchman's stable; it need not be stated of the gallant nation, that it was given up with the greatest pleasure and promptitude. One thing only was lacking to make the resemblance complete and that was a white spot, with the ex-empress used to amuse herself by trying to cover it with a small silver coin: a French artist engaged to make the hairs white at the spot, and while he was performing his task, another medical man of very great eminence proposed an opposite plan of treatment, which was to shock her back to intelligence, by placing her in circumstances of calm quiet and then by announcing the great loss she has sustained in the tragical fate of her husband, and summons to her fortitude, and to stand up to the trials of the hour.

On general principles the latter plan is the best, because the best way to meet any trouble is to stand up to it and look it fully in the face; the mind is thus often brought to bay and in the desperation of the moment hews its way through obstacles and discouragements and stands out disenthralled—its own master once again. This way of running away from trouble or overwhelming calamity is a cowardice and a folly, equal to that of the poor bird which feels itself safe if it can only poke its head in a hole. Let storm be met with defiance; and if a world of troubles come at once, single out one, dispose of it; then another and another, and thus vanquish them in detail.

Many a weak-minded man quails under pecuniary difficulties and seeks deliverence in suicide, thus leaving his poor and faithful wife to battle with difficulties alone which, with a brave joint effort, might have been happily overcome.

